# The Support Person's Preferences and Perspectives of Physical Activity Programs for Older Adults With Cognitive Impairment

**DOI:** 10.3389/fpubh.2021.704561

**Published:** 2021-09-23

**Authors:** Terence W. H. Chong, Emily You, Kathryn A. Ellis, Kay L. Cox, Karra D. Harrington, Stephanie R. Rainey-Smith, David Ames, Nicola T. Lautenschlager, AIBL Research Group

**Affiliations:** ^1^Academic Unit for Psychiatry of Old Age, Department of Psychiatry, The University of Melbourne, Melbourne, VIC, Australia; ^2^St Vincent's Hospital Melbourne, Fitzroy, VIC, Australia; ^3^Epworth Healthcare, Melbourne, VIC, Australia; ^4^North Western Mental Health, Royal Melbourne Hospital, Parkville, VIC, Australia; ^5^Florey Institute of Neuroscience and Mental Health, Parkville, VIC, Australia; ^6^Royal Perth Hospital Unit, Medical School, University of Western Australia, Perth, WA, Australia; ^7^Centre for Healthy Aging, The Pennsylvania State University, University Park, PA, United States; ^8^Centre of Excellence for Alzheimer's Disease Research and Care, School of Medical and Health Sciences, Edith Cowan University, Joondalup, WA, Australia; ^9^Sir James McCusker Alzheimer's Disease Research Unit, Hollywood Private Hospital, Nedlands, WA, Australia; ^10^National Ageing Research Institute, The University of Melbourne, Melbourne, VIC, Australia; ^11^Division of Psychiatry and WA Centre for Health and Ageing, University of Western Australia, Perth, WA, Australia

**Keywords:** support persons, Alzheimer's disease, exercise, physical activity, mild cognitive impairment, carers

## Abstract

**Objectives:** Physical activity (PA) is beneficial for older adults' cognition. There is limited research investigating perspectives of support persons (SPs) of next-of-kins (NOKs) with cognitive impairment. This exploratory study aimed to investigate perspectives of SPs of older adults with Alzheimer's Dementia (AD) or Mild Cognitive Impairment (MCI).

**Methods:** A telephone survey of 213 SPs of NOKs from the Australian Imaging, Biomarkers and Lifestyle Flagship Study of Ageing (AIBL) was undertaken to quantitatively assess SPs' beliefs and knowledge about PA benefits, current PA level of their NOK, and PA program preferences. The contribution of age, gender, diagnosis and mental health symptoms was assessed using multiple logistic regression analyses.

**Results:** Many SPs were aware of PA benefits for memory (64%) and believed it would help their NOK (72%). Older SP age was associated with less awareness of benefits (*p* = 0.016). SPs caring for male NOKs were more likely to believe that PA would be helpful than those caring for female NOKs (*p* = 0.049). NOK AD diagnosis (rather than MCI) (*p* = 0.014), older age (*p* = 0.005) and female gender (*p* = 0.043) were associated with lower PA levels. SPs were mixed regarding preference for their NOKs to participate in individual (45%) or group (54%) PA. Many SPs wanted to participate in PA with their NOK (63%).

**Conclusions:** The results highlight that SPs have high levels of awareness of the cognitive benefits of PA, and describe their preferences regarding PA programs. The findings provide new information to inform targeted public health messaging, PA prescribers and providers, and future research directions.

## Introduction

The prevalence of dementia continues to increase worldwide (1). Mild Cognitive Impairment (MCI) is an intermediate state between healthy ageing and early dementia, where individuals have objective cognitive impairment without functional impairment (2). Having MCI increases an individual's risk of developing dementia (3).

The absence of effective disease modifying treatments for dementia adds even more importance to addressing modifiable risk factors (4). Physical inactivity is the single most significant modifiable risk factor in the USA, UK, Europe and Australia (5, 6).

Physical activity (PA) interventions have been incorporated into guidelines for dementia prevention and management of MCI, including guidelines from the World Health Organization (7), the American Academy of Neurology (8), and the Australian Physical Activity Guidelines for Older Australians with MCI and Subjective Cognitive Decline (9, 10). Evidence from meta-analyses suggests that PA interventions are beneficial to cognitive function in individuals with dementia (11), and Alzheimer's dementia (AD) (12, 13).

Despite the benefits of PA for cognition and general health (14), 65% of Australian older adults are insufficiently active (15). Furthermore, individuals with MCI or dementia have lower PA levels and higher sedentary behaviour levels (16, 17). This indicates a significant research translation gap between evidence for the benefits of PA for individuals with MCI and AD and actual PA levels being undertaken.

An important part of addressing this research gap is to ascertain the preferences and perspectives of older adults, particularly those with cognitive impairment, and their support persons (SPs) toward PA programs. In this study, the term “support person” has been used rather than “carer” as people with MCI often do not need a “carer.” SPs undertake a critical role in the well-being of people with cognitive impairment. This role is emphasised by older adults with cognitive impairment identifying “lack of companion” as a barrier to undertaking PA (18), as well as spouses of individuals with AD expressing concern about leaving their partner alone (19). Furthermore, research into determinants of PA level in people with AD showed that the SPs' “perceived benefit” and “outcome expectation” of PA partially mediated the reported level of PA of their NOK (20). Adding further weight to this, a scoping study noted that the impact of PA programs on SPs is a neglected issue that needs to be considered in optimising these programs for people with dementia (21). From the perspective of the health of SPs, a number of reviews have reported that PA programs for SPs improve their psychological health (22, 23). All of these findings together highlight the importance of exploring the perspectives of SPs which is a further gap in the research literature.

The aim of the AIBL (Australian Imaging, Biomarkers and Lifestyle Flagship Study of Ageing) Support Person Physical Activity Study was to explore the perspectives of SPs of people with cognitive impairment regarding PA for their next-of-kin (NOK), and whether there were differences between SPs of people with MCI compared to AD. Given the importance of SPs in supporting individuals with cognitive impairment to engage in PA, the study outcomes could help maximise the chance of successful implementation of PA programs for people with cognitive impairment.

Our hypotheses were that:

1. Many SPs will be aware of the cognitive benefits of PA for their NOK.
2. SPs of people with Alzheimer's dementia (SPAD) will be less optimistic than SPs of people with Mild Cognitive Impairment (SPMCI) about PA benefits given the greater cognitive and functional barriers associated with AD (24).
3. PA levels will be higher in NOKs with MCI compared with AD given the greater barriers caused by cognitive and functional impairment from AD (16, 17).
4. Older age and higher NOK levels of anxiety and depression will inversely correlate with reported PA levels given that both are barriers to PA (25–27).
5. SPMCI will be more likely to prefer independent rather than group PA when compared with SPAD given the greater care needs associated with AD. Consistent with gender preference findings in the Fitness for the Ageing Brain Qualitative (FABSQual) study, SPs of female NOKs would prefer group programs and female SPs would be more likely to prefer to engage in PA with their NOK (18).

## Materials and Methods

### Sample

We surveyed SPs supporting NOKs who were participants in AIBL - a large prospective longitudinal study (28). This convenience sample was chosen as there was much available data on SPs and their NOKs, including demographics and NOK cognitive and diagnostic assessments. The AIBL study is an ongoing prospective longitudinal study of more than 1,000 volunteers, aged 60 years and above, with AD, MCI or cognitively healthy. The NOKs in our study were classified at their 18-month AIBL assessment with a diagnosis of AD using NINCDS-ADRDA criteria (29) or MCI using criteria described by Winblad and colleagues (30). Diagnostic classification was determined via neuropsychological testing and a clinical review panel (28). The NOKs were from the AIBL cohort, and the original AIBL exclusion criteria included age <60 years, a history of non-AD dementia, schizophrenia, bipolar disorder, significant current (but not past) depression, Parkinson's disease, cancer within the last two years, head injury with >1 hour post-traumatic amnesia, symptomatic stroke, uncontrolled diabetes, obstructive sleep apnea, or current regular alcohol use exceeding two standard drinks per day for women or four per day for men (28).

In the AIBL study, participants nominated an informant or next of kin when they joined the study, This nominated person is the SP that we invited to participate in our survey. The opinions of SPs of NOKs in the AIBL cohort with cognitive impairment were sought through a quantitative telephone survey. This survey used similar questions to the FABSQual study, which involved focus groups and interviews of individuals with and without cognitive impairment (18). Remaining data were sourced from the AIBL database at the time point closest to this survey – the 18-month assessment.

There were 277 NOKs in the AIBL cohort with cognitive impairment (196 with AD and 81 with MCI) at their 18-month AIBL assessment. All 277 NOKs had SPs, and of these, 213 SPs completed the survey.

These data were collected as part of the AIBL study, which was approved by the institutional ethics committees of Austin Health, St Vincent's Health, Hollywood Private Hospital, and Edith Cowan University. All volunteers provided written informed consent before participating in the study.

### Survey Questions and Variables

There were four survey questions (Table 1). The outcome variables of the current study were derived from these questions.

**Table 1:** Telephone interview questions asked of SPs of NOKs with MCI or AD.

**No**.	**Question**
1.	Have you heard about the research that showed that physical activity may help with memory problems? Yes No
2.	Do you think that physical activity will be of benefit to your next of kin? Yes No Don't Know
3a.	How much physical activity or exercise did your next of kin do in the last week. I - sedentary (no minutes of physical activity) II - <150 min (2.5 h) of moderate intensity e.g., brisk walking III - 150 min (2.5 h) or more of moderate and/or vigorous physical activity
3b.	If applicable, what type of physical activity or exercise does your next of kin currently do? Record a list
4a.	Would you prefer physical activity for your next of kin to be conducted in an organised group program or independently in your own environment? Organised Group Independent/Own Environment
4b.	Would you like to participate in physical activity with your next of kin? Yes No Don't Know

The predictor variables for these outcomes included in the logistic regression models were NOK factors – diagnosis (MCI or AD), age, gender, Hospital Anxiety and Depression Scale (HADS)-depression score, HADS-anxiety score and SP factors – age, gender. The HADS is a validated and reliable self-report measure of anxiety and depression comprising seven anxiety and seven depression items, each rated from 0 to 3 points (31). PA levels were classified using the answer to survey question 3a (see Table 1) with “sedentary” referring to no minutes of PA in the past week, “insufficiently active” as <150 min of PA of at least moderate intensity, and “sufficiently active” as at least 150 min of PA of at least moderate intensity.

### Data Analysis

In the dataset, there were missing values for four of the seven variables (see Table 2). The proportion of missing values for these four variables were 21% for HADS–anxiety and depression, 9.9% for SP gender, and 16% for SP age. Applying Little's MCAR (missing completely at random) test to the dataset showed that data was not missing completely at random (X^2^ = 28.8, *p* = 0.00). This was consistent with our observations as to the reasons for the missing data not being random. The missing HADS–anxiety and depression data were most commonly due to NOKs having cognitive impairment that resulted in them being unable to complete this self-report scale - 161 NOKs in our sample had AD and 52 had MCI. The most common reason for missing SP gender and age data was because their NOK was in a residential aged care facility (RACF) and thus, the SP survey respondent may have been a professional carer rather than an SP whose demographic data is collected. This means that missing data were more likely to be from NOKs with more severe cognitive impairment and NOKs needing to live in RACFs.

**Table 2:** Descriptive analysis of NOK and SP characteristics and outcomes by AD and MCI diagnoses.

	**AD**	**MCI**	**Total**		
	***n*** **(%) or mean/SD** (***n***)	***n*** **(%) or mean/SD** (***n***)	***n*** **(%) or mean/SD** (***n***)	**Chi-square or** ***t*****-test**	***p***
SP age (*n* = 179)	71.7/11.0 (135)	69.8/12.1 (44)	71.2/11.3	0.27	0.33
SP gender (male) (*n* = 192)	57 (39.3)	12 (25.5)	69 (35.9)	2.93	0.087
NOK age (*n* = 213)	76.5/7.9 (161)	74.3/7.1 (52)	76.0/7.7	0.91	0.071
NOK gender (male) (*n* = 213)	69 (42.9)	28 (53.8)	97 (45.5)	1.91	0.17
NOK HADS-depression (*n* = 168)	4.1/3.8 (119)	3.7/2.5 (49)	4.0/3.5	4.32	0.54
NOK HADS-anxiety (*n* = 168)	4.2/3.6 (119)	4.3/3.1 (49)	4.3/3.5	0.56	0.84
**Outcome measures (*****n*** **=** **213)**	***n*** **(%)**	***n*** **(%)**	***n*** **(%)**		
**Q1: Have you heard about the memory benefit of PA as shown in research? (Yes)**	104 (64.6)	33 (63.5)	137 (64.3)		
**Q2: Do you think PA is beneficial to your NOK? (Yes)**	115 (71.4)	38 (73.1)	153 (71.8)		
**Q3a: How much PA did your NOK do in the last week?**					
Sedentary (no minutes of PA)	46 (28.6)	5 (9.6)	51 (23.9)		
<150 min of moderate intensity, e.g., Brisk walking	66 (41.0)	22 (42.3)	88(41.3)		
150 min or more of moderate and?/or vigorous PA	49 (30.4)	25 (48.1)	74(34.7)		
**Q3b: Types of PA activities**					
No physical activity	38 (23.6)	3 (5.8)	41(19.2)		
Walking/Unspecified	107 (66.5)	38 (73.1)	145 (68.1)	0.79	0.37
Gardening/Housework/Farming	19 (11.8)	10 (19.2)	29 (13.6)	1.85	0.17
Formal class/Gym/Dance/Strength/Balance	28 (17.4)	10 (19.2)	38 (17.8)	0.091	0.76
Racquet sports/Bowls/Golf/Croquet	15 (9.3)	12 (23.1)	27 (12.7)	6.72	0.010*
Running/Cycling/Aquatics/Rowing	11 (6.8)	7 (13.5)	18 (8.5)	2.23	0.14
**Q4a: Would you prefer group or independent PA?**					
Prefer group activity (vs. others)	90 (55.9)	25 (48.1)	115 (54.0)		
Prefer independent activity (vs. others)	67 (41.6)	28 (53.8)	95 (44.6)		
**Q4b: Would you like to participate in PA with your NOK? (Yes)**	101 (62.7)	33 (63.5)	134 (62.9)		

*if not specified, the sample size for the variable is 213*.

* *p < 0.05*.

To address missing data, we firstly performed multiple imputation. Based on the recommendations of Jakobsen and colleagues (32), the magnitude of missingness in our data was below 40% and multiple imputation was therefore appropriate to use. Specifically, we included all predictor and outcome variables of interest (see Table 2) in the imputation model, selected “Automatic” imputation method, and set the number of imputations as 25 [the minimum number of imputations against the highest proportion of missing values for the HADS-anxiety and depression variables (21%)] (33). We also set constraints for all continuous variables with missing values (e.g., minimum SP age set at 18 years and the range of HADS-anxiety and depression set at 0–21) to ensure that imputed values for these variables would be within the normal range.

Mean values (SDs) and frequencies (percentages) were calculated for continuous and categorical variables, respectively. *T*-tests and chi-square tests were conducted as necessary to compare differences in these variables between SPAD and SPMCI as well as NOKs with AD and MCI. To identify significant predictors of the outcomes, we conducted multivariable logistic regression analyses for each outcome variable using the imputed datasets. Odds ratios (OR) were calculated for each predictor in each model as a measure of effect size. We also performed the same analyses for each outcome variable using the original dataset with missing data (see Supplementary Material). Given that missing data can lead to the loss of power and potentially biassed results (33), unless specified, we discussed the results of the imputed datasets.

Collinearity of all predictor variables was tested using the linear regression procedure. Statistical significance was assessed using *p* < 0.05 and 95% confidence interval (CI) in logistic regression models. ORs were used to quantify the association between predictor and outcome variables. All analyses were performed in SPSS 24 (34).

## Results

The descriptive analysis of participant characteristics and outcomes overall, and by AD and MCI diagnoses, are presented in Table 2. The mean age of SPs was 71 years and 64% were female. The NOKs had a mean age of 76 years, 54% were female, 75% had a diagnosis of AD and 25% MCI. NOKs had mean scores on the HADS-anxiety and depression subscales that are indicative of subthreshold levels of depression and anxiety, and there was no significant difference between NOKs with AD and NOKs with MCI.

**Questions 1 and 2. “Have you heard about the memory benefit of physical activity (PA) as shown in research?” And “Do you think PA is beneficial to your NOK?”**

The majority of SPs (64%) had heard about the memory benefit of PA as shown in research and a greater majority (72%) thought that PA would be beneficial to their NOK.

In the logistic regression analysis shown in Table 3, older SPs were less likely to report that they had heard of the benefit of PA as shown in research (OR = 0.95; 95% CI: 0.92–0.99; *p* = 0.016). In addition, SPs of male NOKs were more likely to believe that PA would be beneficial to their NOK (OR = 2.90; 95% CI: 1.00–8.41; *p* = 0.049), however this was not statistically significant in the original dataset analysis.

**Table 3:** Results of logistic regression analyses of NOK characteristics and outcomes.

**Factors**	**Imputed Dataset**	
	**B (95% CI)**	***P***
**Question 1**		
NOK Category (AD vs. MCI)	1.17 (0.58–2.35)	0.66
NOK Age	0.98 (0.93–1.03)	0.38
NOK Gender	2.01 (0.71–5.65)	0.19
SP Age	0.95 (0.92–0.99)	0.016*
SP Gender	2.49 (0.76–8.19)	0.13
NOK HADS depression	1.04 (0.92–1.17)	0.54
NOK HADS anxiety	0.92 (0.82–1.04)	0.18
**Question 2**		
NOK Category (AD vs. MCI)	1.17 (0.55–2.47)	0.68
NOK Age	0.96 (0.92–1.01)	0.12
NOK Gender	2.90 (1.00–8.41)	0.049*
SP Age	0.98 (0.94–1.02)	0.24
SP Gender	1.39 (0.43–4.45)	0.58
NOK HADS depression	0.97 (0.85–1.10)	0.62
NOK HADS anxiety	1.06 (0.92–1.21)	0.44
**Question 4a Group**		
NOK Category (AD vs. MCI)	1.24 (0.65–2.39)	0.52
NOK Age	1.02 (0.98–1.06)	0.40
NOK Gender	1.58 (0.61–4.06)	0.35
SP Age	0.98 (0.94–1.01)	0.18
SP Gender	2.83 (0.96–8.38)	0.060
NOK HADS depression	1.08 (0.97–1.22)	0.18
NOK HADS anxiety	0.99 (0.88–1.12)	0.92
**Question 4a Independent**		
NOK Category (AD vs. MCI)	0.74 (0.38–1.43)	0.36
NOK Age	0.96 (0.92–1.01)	0.092
NOK Gender	0.89 (0.36–2.21)	0.80
SP Age	1.01 (0.97–1.04)	0.74
SP Gender	0.40 (0.14–1.12)	0.082
NOK HADS depression	0.92 (0.81–1.04)	0.16
NOK HADS anxiety	1.00 (0.89–1.13)	1.00
**Question 4b**		
NOK Category (AD vs. MCI)	0.92 (0.47–1.82)	0.82
NOK Age	1.00 (0.96–1.05)	0.84
NOK Gender	1.02 (0.41–2.55)	0.96
SP Age	1.02 (0.98–1.05)	0.38
SP Gender	1.34 (0.49–3.71)	0.57
NOK HADS depression	0.97 (0.87–1.09)	0.62
NOK HADS anxiety	0.94 (0.84–1.06)	0.32

* *p < 0.05*.

**Questions 3a and b: “How much PA did your NOK do in the last week?” And “Which type of PA does your next of kin currently do?”**

Overall, there was no statistically significant difference between the level of PA being performed by NOKs in this survey compared with the general population in Australia aged 65 years and above, as shown in Table 4 (X^2^ = 2.33, *p* = 0.31) (15).

**Table 4:** Comparison of PA level of NOKs in this study with adults aged over 65 years in the general population in Australia (15).

	**AD (%)**	**MCI (%)**	**Total (%)**	**General Australian population aged 65 years and over in 2014–15 (%)**
**Q3a: How much PA did your NOK do in the last week?**				
Sedentary (no minutes of PA)	29	10	24	28
<150 min of moderate intensity	41	42	41	37
150 min or more of moderate and/or vigorous PA	30	48	35	35

NOKs in both the AD and MCI groups undertook a broad range of PA types with walking being the most popular. A larger proportion of the MCI group (23%) compared to the AD group (9%) were involved in formal sport including racquet sports, bowls, golf and croquet, while there were no large differences for our other categories of PA - walking/unspecified, gardening/housework/farming, formal classes/gym/dance/strength/balance and running/cycling/aquatics/rowing.

The results of the logistic regression analysis of level of PA are summarised in Table 5. When comparing PA level of sedentary with insufficiently active (<150 min PA/week), there were no statistically significant findings.

**Table 5:** Results of logistic regression analyses of PA level of NOK.

**Factors**	**Imputed Dataset**	
	**B (95% CI)**	***p***
**Question 3a sedentary vs**. **<150 min PA/week**		
NOK Category (AD vs. MCI)	0.35 (0.12–1.01)	0.052
NOK Age	0.97 (0.81–1.02)	0.23
NOK Gender	2.32 (0.72–7.43)	0.18
SP Age	1.00 (0.95–1.04)	0.82
SP Gender	1.92 (0.53–6.92)	0.32
NOK HADS depression	1.01 (0.87–1.18)	0.87
NOK HADS anxiety	1.01 (0.87–1.18)	0.86
**Question 3a sedentary vs. ≥150 min PA/week**		
NOK Category (AD vs. MCI)	0.25 (0.085–0.76)	0.014*
NOK Age	0.91 (0.86–0.97)	0.005**
NOK Gender	3.81 (1.04–13.9)	0.043*
SP Age	0.99 (0.95–1.04)	0.80
SP Gender	1.94 (0.46–8.10)	0.36
NOK HADS depression	0.95 (0.81–1.13)	0.57
NOK HADS anxiety	1.08 (0.91–1.27)	0.38

* *p < 0.05*.

** *p < 0.01*.

When comparing PA level of sedentary with being sufficiently active (≥150 min PA/week), male gender of NOK was associated with being more physically active (OR = 3.81; 95% CI: 1.04–13.9; *p* = 0.043). NOKs with a diagnosis of AD (OR = 0.25; 95% CI = 0.085–0.76; *p* = 0.014) and NOKs who were older (OR = 0.91; 95% CI = 0.86–0.97; *p* = 0.005) were less likely to be sufficiently active and more likely to be sedentary. In the original dataset analysis, the association was only statistically significant for age of NOK.

**Questions 4a and b: “Would you prefer group or independent PA?” And “Would you like to participate in PA with your next of kin?”**

The responses of SPs to these questions were mixed. Approximately half of the SPs preferred group activity or both (54%), and approximately half preferred individual activity or both (45%), with others answering with no preference or “don't know”. Almost two-thirds of the SPs reported that they would like to participate in PA with their NOK (63%).

In the logistic regression shown in Table 3, there were no factors that were statistically significant in predicting a preference toward individual PA or group PA.

## Discussion

Our survey results support the hypothesis that many SPs had heard about research showing the benefits of PA for memory (64%) and believe it would be beneficial to their NOK (72%). This finding provides new information about beliefs of SPs which have not previously been investigated, and contrasts with qualitative research findings that participants did not express beliefs about benefits of PA for AD (19). Our findings are encouraging as they suggest that information about the benefits of PA for memory is being received by the public. This may be due to public health campaigns, health professionals or the media. Our results also complement the findings of the FABSQual study which showed that older adults with cognitive impairment had a positive attitude toward PA and believed it was beneficial to cognition (18).

We found that younger SPs were more likely to be familiar with the research findings regarding memory benefits of PA, and SPs of male NOKs were more likely to believe that PA would be beneficial. The latter might be related to the finding that male NOKs were more physically active than female NOKs, both in this study and the general population (25, 35). The results of our survey did not support our hypothesis that SPAD would be less optimistic than SPMCI about the benefits of PA for their NOK.

Our hypothesis that NOKs with MCI would be more physically active than the NOKs with AD was supported, which is consistent with previous research (16, 17). Regarding type of PA, a higher proportion of NOKs with MCI than AD were involved in formal sport such as racquet sports, bowls, golf and croquet. This may reflect the greater cognitive demands of such activities when compared with other activities and is consistent with the previous finding that older adults with cognitive impairment prefer “simple/light/safe” activities (18).

Male gender and younger age of NOKs were associated with being more physically active, consistent with previous research (20, 25, 35). The results of our survey did not support the hypothesis that increasing NOK anxiety and depression scores would inversely correlate with reported levels of PA. This may reflect the AIBL exclusion criteria relating to higher levels of depressive symptoms (28).

There was quite an even split of SP preferences for group or individual PA for their NOK and no factors significantly predicted these preferences. Thus, we could not support our hypotheses relating to these preferences. This diversity of preference is consistent with previous research findings that SPs and NOKs prefer PA programs tailored to the individual (18, 19). Almost two-thirds of SPs (63%) expressed that they would like to participate in PA with their NOKs. This is an encouraging finding, both given that participants with cognitive impairment have cited “lack of companion” as a barrier to PA (18), and that PA interventions for carers may reduce subjective caregiver burden (36).

Of note, the diagnostic category of the NOK had less of an effect on the survey responses of SPs than we had hypothesised, with differences only found for the questions about amount and type of PA undertaken by their NOK. This finding has implications as to how to optimally engage with SPs regarding PA for their NOK. SPs in this study were aware of their NOK having a diagnosis of AD and what this meant, as the AIBL researchers informed participants, NOK and treating clinicians of this diagnosis to ensure appropriate follow-up. SPs may not be aware of their NOK having MCI, however they would likely be aware of subjective memory complaints as MCI was defined as subjective memory complaints plus objective cognitive testing findings. Given that the questions were about cognition in general and about their NOK specifically, it is expected that knowledge of diagnostic category would not have a significant influence on the results.

Study limitations included convenience sampling from AIBL limiting the generalizability of findings. This is because AIBL inclusion and exclusion criteria are relatively strict, meaning that NOKs surveyed do not have major mental health conditions, and are more educated and physically active than the general population (28). This survey provides preliminary data from a large sample to inform future qualitative research. There was also some missing data in several measurements. Another limitation is the potential for questionnaire bias through the use of leading questions, and results need to be interpreted in this context. We would expect some generalizability of predictors of PA level to international settings, although awareness, beliefs and preferences regarding PA may vary.

An important strength of the study is that it is among the first to survey SPs of people with MCI and AD regarding their views about PA. We can also be confident about NOKs' diagnostic classification of MCI and AD due to the rigour of AIBL assessments. Moreover, the sample size is relatively large for a study of this population type.

Our findings provide encouragement that SPs have some awareness of the benefit of PA for memory. SPs have an important role to play in their NOK undertaking PA and thus the bridging of this research translation gap. The findings also suggest that public health campaign messages may be helpful, and that older SPs and those caring for female NOKs, could be specifically targeted. The findings also provide guidance to service providers to include both individual and group-based PA programs, as well as the option for SPs to participate. Overall, the results suggest that there is much potential benefit from engaging with support persons when offering PA interventions, given that many have some awareness of its benefits, and moreover, believe it may be beneficial to their NOK. It appears important that interventions include multiple options that can be tailored to the individual and their SP, and that preferences may vary as a result of the age and gender of SP and NOK, the diagnostic category of their NOK, and preferences such as more “formal sport” for NOKs with MCI compared to “simpler” activities for NOKs with AD. Interventions could also incorporate recently published PA guidelines for older adults with MCI or SCD (9, 10). Given that there is already awareness of the benefits of PA, future research could include qualitative research into the preferences and perspectives of SPs toward PA. This could particularly focus on the research translation gap given that in many parts of the world, there are low rates of older adults meeting PA guideline recommendations, despite knowledge about the benefits of PA. It would be critical to explore barriers and enablers of physical activity, and translational research around dissemination and implementation of PA guidelines, including behaviour change interventions to increase motivation and adherence to PA programs.

## Data Availability Statement

The datasets presented in this article are not readily available because they are part of the AIBL dataset, whereby applications for access can be made via an Expression of Interest process. Requests to access the datasets should be directed to https://aibl.csiro.au/research/support/.

## Ethics Statement

The studies involving human participants were reviewed and approved by Austin Health, St Vincent's Health, Hollywood Private Hospital, and Edith Cowan University. The participants provided their written informed consent to participate in this study.

## Author Contributions

TC, KE, KC, KH, and NL contributed to study design. KE, KH, SR-S, DA, NL, and the AIBL Research Group contributed to data collection. EY and TC contributed to data analysis. All authors contributed to the article and approved the submitted version.

## Funding

This work was supported by funding from Pfizer/Wyeth. The funder was not involved in the study design, collection, analysis, interpretation of data, the writing of this article or the decision to submit it for publication.

## Conflict of Interest

The authors declare that the research was conducted in the absence of any commercial or financial relationships that could be construed as a potential conflict of interest.

## Publisher's Note

All claims expressed in this article are solely those of the authors and do not necessarily represent those of their affiliated organizations, or those of the publisher, the editors and the reviewers. Any product that may be evaluated in this article, or claim that may be made by its manufacturer, is not guaranteed or endorsed by the publisher.
