# Supplementary material for: The Support Person's Preferences and Perspectives of Physical Activity Programs for Older Adults With Cognitive Impairment
Source: Front Public Health. 2021 Sep 23;9:704561. doi: 10.3389/fpubh.2021.704561 (PMC8495209; doi:10.3389/fpubh.2021.704561)
Supplement: Supplementary file 1 [file Table_1.DOCX]

**Title: The Support Person’s Preferences and Perspectives of Physical Activity Programs for Older Adults With Cognitive Impairment**

**Supplementary Material**

Table S1. Results of logistic regression analyses of NOK characteristics and outcomes

| **Factors** | **Imputed Dataset** | | **Original Dataset** | |
| --- | --- | --- | --- | --- |
|  | **B (95% CI)** | **p** | **B (95% CI)** | **p** |
| **Question 1** |  |  |  |  |
| NOK Category (AD vs MCI) | 1.17 (0.58-2.35) | 0.66 | 1.31 (0.58-3.00) | 0.51 |
| NOK Age | 0.98 (0.93-1.03) | 0.38 | 0.97 (0.91-1.03) | 0.33 |
| NOK Gender | 2.01 (0.71-5.65) | 0.19 | 2.79 (0.66-11.0) | 0.17 |
| **SP Age** | **0.95 (0.92-0.99)** | **0.016*** | **0.94 (0.89-0.99)** | **0.012*** |
| SP Gender | 2.49 (0.76-8.19) | 0.13 | 2.73 (0.57-13.2) | 0.21 |
| NOK HADS depression | 1.04 (0.92-1.17) | 0.54 | 1.09 (0.94-1.26) | 0.28 |
| NOK HADS anxiety | 0.92 (0.82-1.04) | 0.18 | 0.96 (0.83-1.11) | 0.57 |
|  |  |  |  |  |
| **Question 2** |  |  |  |  |
| NOK Category (AD vs MCI) | 1.17 (0.55-2.47) | 0.68 | 1.17 (0.48-2.85) | 0.74 |
| NOK Age | 0.96 (0.92-1.01) | 0.12 | 0.95 (0.88-1.12) | 0.14 |
| **NOK Gender** | **2.90 (1.00-8.41)** | **0.049*** | 2.49 (0.58-10.7) | 0.22 |
| SP Age | 0.98 (0.94-1.02) | 0.24 | 0.97 (0.92-1.01) | 0.16 |
| SP Gender | 1.39 (0.43-4.45) | 0.58 | 0.96 (0.20-4.55) | 0.96 |
| NOK HADS depression | 0.97 (0.85-1.10) | 0.62 | 0.94 (0.80-1.09) | 0.39 |
| NOK HADS anxiety | 1.06 (0.92-1.21) | 0.44 | 1.15 (0.97-1.35) | 0.11 |
|  |  |  |  |  |
| **Question 4a Group** |  |  |  |  |
| NOK Category (AD vs MCI) | 1.24 (0.65-2.39) | 0.52 | 1.03 (0.49-2.18) | 0.94 |
| NOK Age | 1.02 (0.98-1.06) | 0.40 | 0.98 (0.92-1.04) | 0.42 |
| NOK Gender | 1.58 (0.61-4.06) | 0.35 | 1.03 (0.31-3.46) | 0.96 |
| SP Age | 0.98 (0.94-1.01) | 0.18 | 0.99 (0.95-1.03) | 0.59 |
| SP Gender | 2.83 (0.96-8.38) | 0.060 | 2.06 (0.53-7.98) | 0.30 |
| NOK HADS depression | 1.08 (0.97-1.22) | 0.18 | 1.14 (0.99-1.32) | 0.065 |
| NOK HADS anxiety | 0.99 (0.88-1.12) | 0.92 | 0.94 (0.83-1.07) | 0.36 |
|  |  |  |  |  |
| **Question 4a Independent** |  |  |  |  |
| NOK Category (AD vs MCI) | 0.74 (0.38-1.43) | 0.36 | 0.88 (0.41-1.85) | 0.73 |
| NOK Age | 0.96 (0.92-1.01) | 0.092 | 0.98 (0.93-1.04) | 0.57 |
| NOK Gender | 0.89 (0.36-2.21) | 0.80 | 1.10 (0.33-3.67) | 0.87 |
| SP Age | 1.01 (0.97-1.04) | 0.74 | 0.99 (0.95-1.03) | 0.64 |
| SP Gender | 0.40 (0.14-1.12) | 0.082 | 0.51 (0.13-1.92) | 0.32 |
| NOK HADS depression | 0.92 (0.81-1.04) | 0.16 | 0.93 (0.81-1.06) | 0.28 |
| NOK HADS anxiety | 1.00 (0.89-1.13) | 1.00 | 1.01 (0.89-1.15) | 0.87 |
|  |  |  |  |  |
| **Question 4b** |  |  |  |  |
| NOK Category (AD vs MCI) | 0.92 (0.47-1.82) | 0.82 | 0.77 (0.34-1.74) | 0.53 |
| NOK Age | 1.00 (0.96-1.05) | 0.84 | 1.01 (0.95-1.07) | 0.80 |
| NOK Gender | 1.02 (0.41-2.55) | 0.96 | 0.46 (0.12-1.79) | 0.27 |
| SP Age | 1.02 (0.98-1.05) | 0.38 | 1.02 (0.97-1.06) | 0.45 |
| SP Gender | 1.34 (0.49-3.71) | 0.57 | 0.50 (0.11-2.19) | 0.36 |
| NOK HADS depression | 0.97 (0.87-1.09) | 0.62 | 0.96 (0.84-1.10) | 0.58 |
| NOK HADS anxiety | 0.94 (0.84-1.06) | 0.32 | 0.95 (0.84-1.09) | 0.48 |

*p<0.05

Table S2. Results of logistic regression analyses of PA level of NOK

| **Factors** | **Imputed Dataset** | | **Original Dataset** | |
| --- | --- | --- | --- | --- |
|  | **B (95% CI)** | **p** | **B (95% CI)** | **p** |
| **Question 3a sedentary vs <150 mins PA/week** |  |  |  |  |
| NOK Category (AD vs MCI) | 0.35 (0.12-1.01) | 0.052 | 0.44 (0.13-1.47) | 0.18 |
| NOK Age | 0.97 (0.81-1.02) | 0.23 | 0.97 (0.89-1.06) | 0.51 |
| NOK Gender | 2.32 (0.72-7.43) | 0.18 | 2.11 (0.39-11.3) | 0.38 |
| SP Age | 1.00 (0.95-1.04) | 0.82 | 0.99 (0.93-1.05) | 0.67 |
| SP Gender | 1.92 (0.53-6.92) | 0.32 | 2.34 (0.36-15.0) | 0.37 |
| NOK HADS depression | 1.01 (0.87-1.18) | 0.87 | 1.04 (0.87-1.23) | 0.68 |
| NOK HADS anxiety | 1.01 (0.87-1.18) | 0.86 | 1.03 (0.86-1.24) | 0.74 |
|  |  |  |  |  |
| **Question 3a sedentary vs ≥150 mins PA/week** |  |  |  |  |
| **NOK Category (AD vs MCI)** | 0.25 (0.085-0.76) | **0.014*** | 0.34 (0.099-1.19) | 0.092 |
| **NOK Age** | 0.91 (0.86-0.97) | **0.005**** | 0.89 (0.91-0.97) | **0.010*** |
| **NOK Gender** | 3.81 (1.04-13.9) | **0.043*** | 1.38 (0.23-8.16) | 0.72 |
| SP Age | 0.99 (0.95-1.04) | 0.80 | 1.00 (0.94-1.06) | 0.91 |
| SP Gender | 1.94 (0.46-8.10) | 0.36 | 0.60 (0.08-4.54 | 0.62 |
| NOK HADS depression | 0.95 (0.81-1.13) | 0.57 | 0.94 (0.77-1.15) | 0.54 |
| NOK HADS anxiety | 1.08 (0.91-1.27) | 0.38 | 1.12 (0.92-1.37) | 0.25 |

*p<0.05

**p<0.01
